# Synthesis of low sidelobe level antenna arrays through only main lobe assumption

**DOI:** 10.1038/s41598-021-01934-8

**Published:** 2021-11-24

**Authors:** Mohammad Khalaj-Amirhosseini

**Affiliations:** School of Electrical Engineering, Iran University of Science and Technology, Tehran, Iran

**Keywords:** Engineering, Electrical and electronic engineering

## Abstract

An analytic method is proposed to design uniformly spaced arrays so that have as low as possible sidelobe level and having directivity as close as to that of uniformly excited arrays. The ideal array factor of arrays is assumed to have only one main lobe. The actual synthesized array would have sidelobe levels which can be controlled by a parameter. Some examples are given to verify the effectiveness of the presented method.

## Introduction

Array factor characteristics of linear and planar antenna arrays are important for many applications such as communication systems, radars and imaging^1^. The sidelobe level (SLL), directivity and beamwidth are three important features of antenna arrays which depend on the excitation currents of the antennas and the distances between them^2,3^.

Uniformly excited arrays of distances equal or more than a half wavelength have the maximum possible directivity. However, the sidelobe level of uniformly excited arrays is high and about − 13.2 dB which makes them less desirable for many applications^2^. Hence, various methods have been presented for sidelobe level reduction by researchers, so far.

Sidelobe reduction by iterative sampling and Fourier transform methods^4,5^, nonuniform distance between the elements^6–9^, self convolution^10^, *m*-th power of uniform array^11^, Fourier method^12^, and some optimization procedures^13–15^ have been studied. In^14^, the arrays are synthesized to have maximum directivity for a specified sidelobe level.

In this paper, we propose an ideal desired array factor which has only a main lobe and has no sidelobes. Indeed, desired array factor is assumed to have only a main lobe. So, one may call this method as Only Main Lobe Assumption (OMLA). This ideal array factor needs infinite number of elements. Therefore, the actual synthesized array would have non zero sidelobes due to truncation of infinite number of elements. The sidelobe level of synthesized array can be controlled by a parameter which is related to the beamwidth of the main lobe of the ideal desired array factor. Unlike some patterns such as Taylor-nbar^2^, the proposed method gives us explicit relations for the excitation currents.

The paper is organized as follows. In “Array factors and excitation currents” section, the relations between the array factors and excitation currents of linear arrays are reviewed. In “Only main lobe assumption” section, the only main lobe assumption (OMLA) method is introduced. In “Verification and comparison” section, the OMLA method is verified using some examples. In “Two dimensional Omla pattern” section, the presented OMLA method is applied for planar arrays by a transformation.

## Array factors and excitation currents

**Figure 1:**
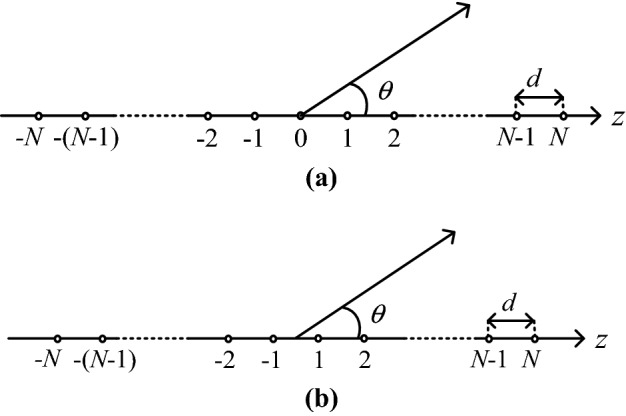
Typical configuration of linear antenna arrays. (**a**) odd number of elements, (**b**) even number of elements.

Figure 1 shows linear antenna arrays having *L* = 2* N* + 1 or *L* = 2* N* elements of equal distances *d* and unequal excitation currents *I*_*n*_. The array factor of linear arrays can be written as follows, for odd and even number of elements, respectively.

1 $$ F(\psi ) = \sum\limits_{n = - N}^{N} {I_{n} exp(jn\psi )} $$

2 $$ F(\psi ) = \sum\limits_{\begin{subarray}{l} n = - N \\ n \ne 0 \end{subarray} }^{N} {I_{n} exp\left( {j(n \mp 0.5)\psi } \right)} $$

where *ψ* is defined as $\psi = 2\pi \frac{d}{\lambda }\cos \theta$ in which *λ* is the wavelength. The upper and lower signs in (2) refers to positive and negative *n*, respectively, in entire this paper.

From the Fourier's series theorem, the excitation currents are related to desired array factor, *F*_*d*_(*ψ*), as follows for odd and even number of elements, respectively.

3 $$ I_{n} = \frac{1}{2\pi }\int_{ - \pi }^{\pi } {F_{d} (\psi )\exp ( - jn\psi )d\psi } $$

4 $$ I_{n} = \frac{1}{2\pi }\int_{ - \pi }^{\pi } {F_{d} (\psi )\exp \left( { - j(n \mp 0.5)\psi } \right)d\psi } $$

## Only main lobe assumption

**Figure 2:**
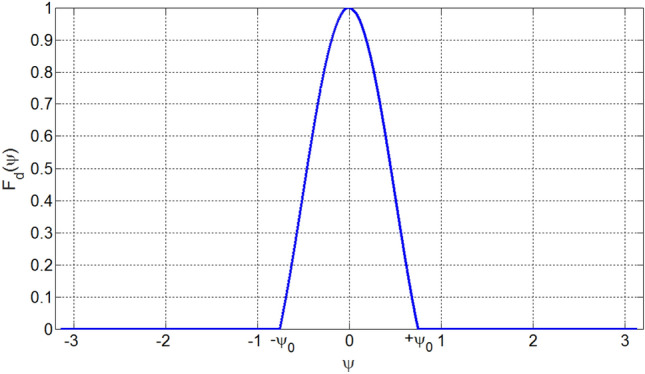
Desired array factor that has only a main lobe of first null beamwidth of 2* ψ*_0_.

We intend to synthesize a linear array which has as low as possible sidelobe level while having directivity as close as to directivity of uniformly excited arrays. To this end, we propose a desired array factor, *F*_*d*_(*ψ*), that has absolutely no sidelobes and has only a main lobe similar to the main lobe of uniformly excited array. Figure 2 shows such a desired array factor that has only a main lobe within the range *ψ* = [− *ψ*_0_ + *ψ*_0_] in which the parameter *ψ*_0_ is an arbitrary value around 2*π*/*L*. Therefore, 2* ψ*_0_ denotes the first null beamwidth (FNBW) of the array factor.

The proposed desired array factor can be designed as the expanded or contracted main lobe of a uniformly excited array. In this way, the desired array factor is written by a summation as follows for odd and even number of elements, respectively.

5 $$  F_{d} (\psi ) = \left\{ {\begin{array}{*{20}l}    {\sum\limits_{{m =  - N}}^{N} {exp(jm\psi /\alpha )} ;} \hfill & { - \psi _{0}  \le \psi  \le \psi _{0} } \hfill  \\    {0;} \hfill & {{\text{otherwise}}} \hfill  \\   \end{array} } \right.  $$

6 $$  F_{d} (\psi ) = \left\{ {\begin{array}{*{20}l}    {\sum\limits_{\begin{subarray}{l}    m =  - N \\    m \ne 0  \end{subarray} }^{N} {exp\left( {j(m \mp 0.5)\psi /\alpha } \right)} ;} \hfill & { - \psi _{0}  \le \psi  \le \psi _{0} } \hfill  \\    {0;} \hfill & {{\text{otherwise}}} \hfill  \\   \end{array} } \right.  $$

In Eqs. (5) and (6), *α* is the expansion factor defined as follows.

7 $$ \alpha = \frac{{\psi_{0} L}}{2\pi } $$

In fact, the expansion factor is the ratio of the width of desired main lobe to the width of the main lobe of uniformly excited array.

Substituting Eqs. (5) and (6) in Eqs. (3) and (4), gives us the required excitation currents as follows for odd and even number of elements, respectively.

8 $$ I_{n} = \frac{{\psi_{0} }}{\pi }\sum\limits_{m = - N}^{N} {{\text{sinc}}\left( {\frac{{\psi_{0} }}{\pi }(m/\alpha - n)} \right)} $$

9 $$ I_{n} = \frac{{\psi_{0} }}{\pi }\sum\limits_{\begin{subarray}{l} m = - N \\ m \ne 0,n \ne 0 \end{subarray} }^{N} {{\text{sinc}}\left( {\frac{{\psi_{0} }}{\pi }[m/\alpha - n + 0.5( \pm 1 \mp 1/\alpha )]} \right)} $$

The first upper and lower signs in Eq. (9) refer to positive and negative *n*, respectively. Also, the second upper and lower signs in Eq. (9) refer to positive and negative *m*, respectively.

It is worth mentioning that the proposed desired array factor is only an assumption for a linear array having definite number of elements. Actually, this array factor can not be realized exactly because it needs infinite number of elements according to Eqs. (8) and (9).

## Verification and comparison

The proposed OMLA method is verified and compared with other methods such as uniform, Chebyshev and Taylor by some examples.

**Figure 3:**
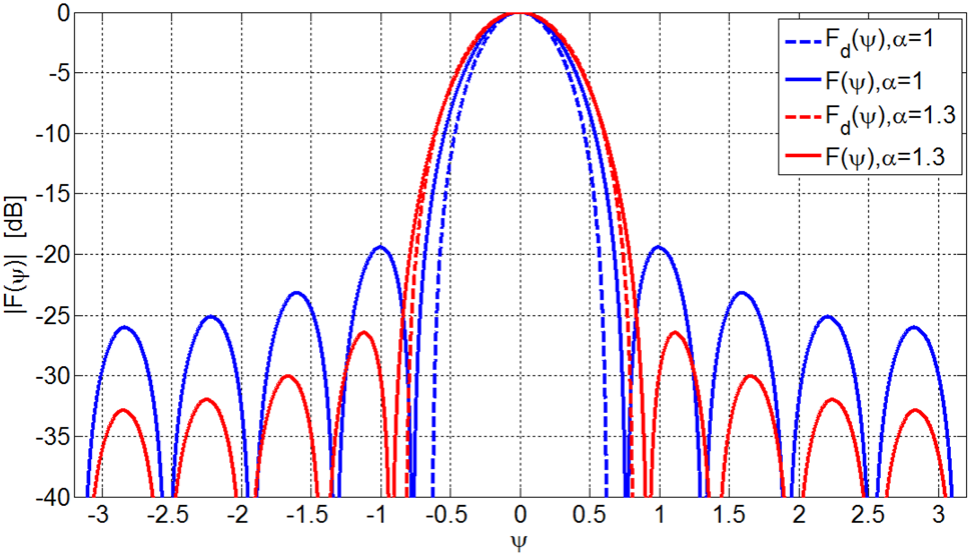
Designed array factors, *F*(*ψ*), for array of *L* = 10 elements.

**Figure 4:**
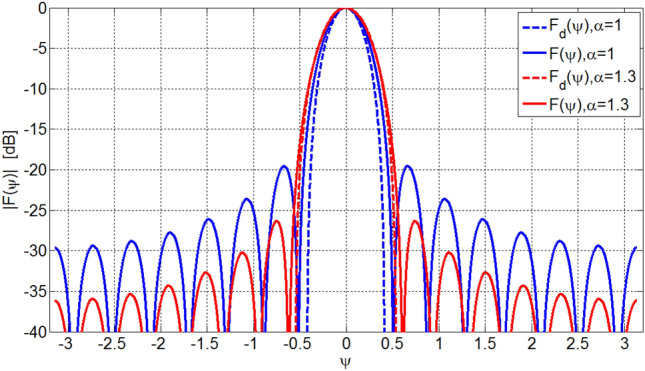
Designed array factors, *F*(*ψ*), for array of *L* = 15 elements.

Two arrays with *L* = 10 and *L* = 15 elements are designed to have the proposed desired array factor. Figures 3 and 4 illustrate four resultant array factors for *α* = 1.0 and 1.3. It is seen that the resultant patterns, *F*(*ψ*), have a main lobe and also several non-zero sidelobes due to truncation of infinite number of elements. The main lobe is somewhat wider than the desired one and its widening is reduced as the expansion factor *α* increases.

**Figure 5:**
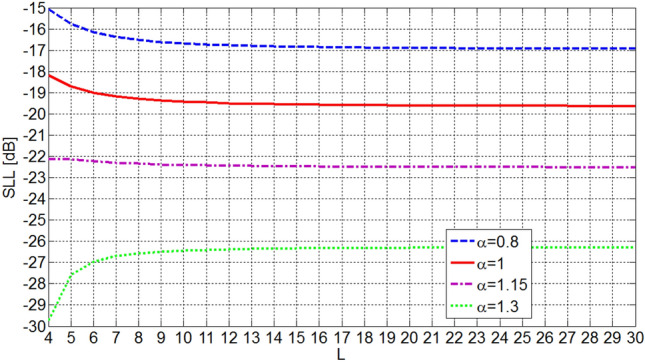
Sidelobe level of designed array versus *L* and *α* as parameter.

**Figure 6:**
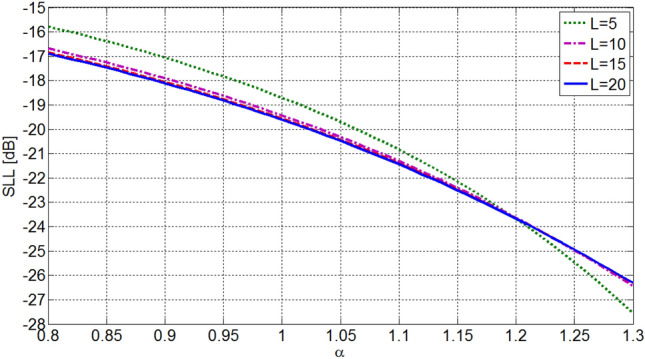
Sidelobe level of designed array versus *α* and *L* as parameter.

The sidelobe level of OMLA patterns are dependent on the expansion factor *α* and the number of elements *L*. Figures 5 and 6 show the dependence of sidelobe level on these parameters. It is seen that the sidelobe level is reduced as the parameter *α* increases and it is almost independent of *L* for *L*s greater than about 8. In fact, as it is seen in Fig. 6, the expansion factor *α* determines SLL. Also, by choosing the number of elements *L* besides *α*, the first null beamwidth of the pattern, i.e. 2* ψ*_0_, is determined by Eq. (7).

**Figure 7:**
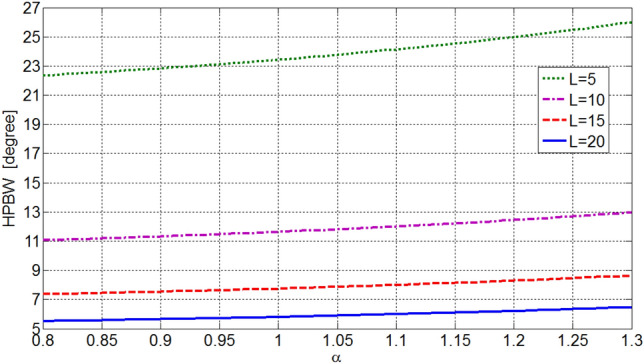
HPBW of designed array versus *α* and *L* as parameter for *d*/*λ* = 0.5.

**Figure 8:**
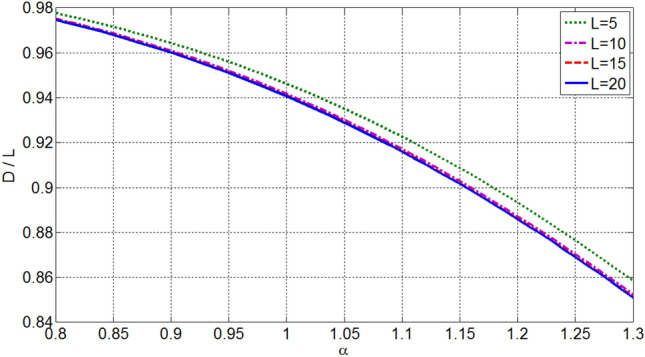
Normalized directivity of designed array versus *α* and *L* as parameter for *d*/*λ* = 0.5.

**Figure 9:**
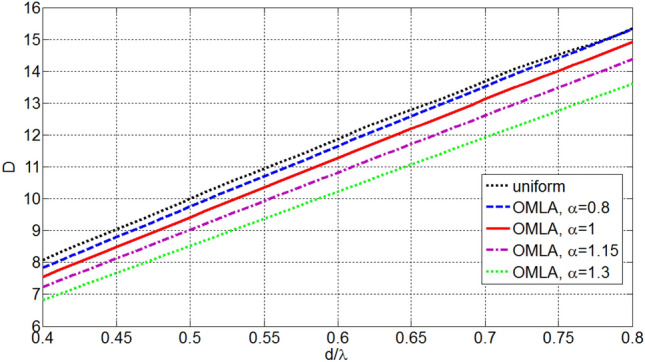
Directivity of designed array versus *d*/*λ* and *α* as parameter for *L* = 10.

The beamwidth and directivity of OMLA patterns are depending on *d*/*λ* as well as *α* and *L*. Figures 7 and 8 illustrate the variation of HPBW and normalized directivity, i.e. *D*/*L*, with respect to *α* and *L* for *d*/*λ* equal to 0.5. It is seen that as the expansion factor *α* increases, the HPBW increases and directivity decreases. So, reduction of SLL is at the expense of some reduction in the directivity, which is expectable in array design. Also, Fig. 9 shows directivity for array of *L* = 10 elements versus *d*/*λ* which indicates increase of directivity as *d*/*λ* increases. One can see from Figs. 8 or 9 and also 6 that the directivity (and HPBW) of OMLA pattern is close to that of uniform array, i.e. *L*, but with SLL less than -19.5 dB rather than being equal to -13.2 dB, for *α*  > 1.

It is worth noting that the proposed OMLA method can be used for synthesizing arrays with supposed radiation characteristics containing SLL and directivity or HPBW. For instance, Figs. 6 and 8 are utilized to obtain the parameter *α* and the number of elements *L* from known SLL and *D*.

**Figure 10:**
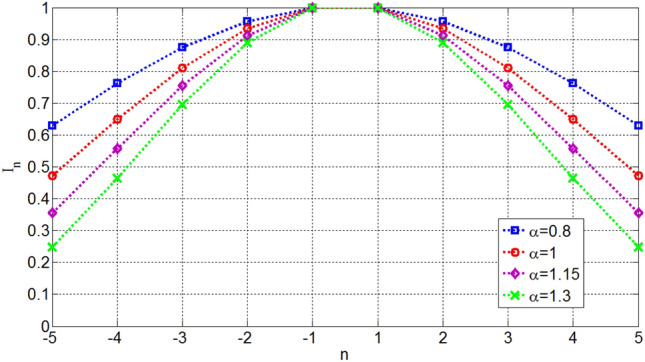
Excitation currents of elements for OMLA pattern of *L* = 10 elements.

**Figure 11:**
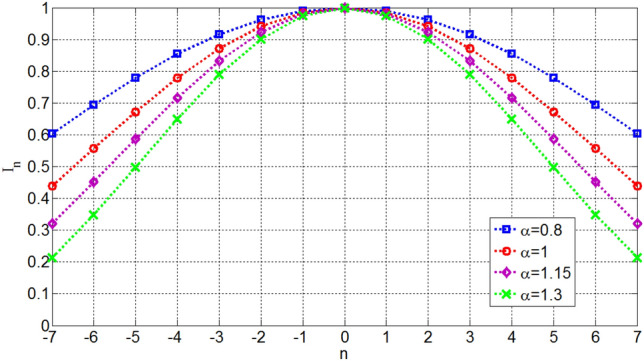
Excitation currents of elements for OMLA pattern of *L* = 15 elements.

Figures 10 and 11 show the required excitation currents of OMLA pattern for some *α*s for *L* = 10 and 15 elements, respectively. It is seen that as *α* increases the tapering of currents increases.

**Figure 12:**
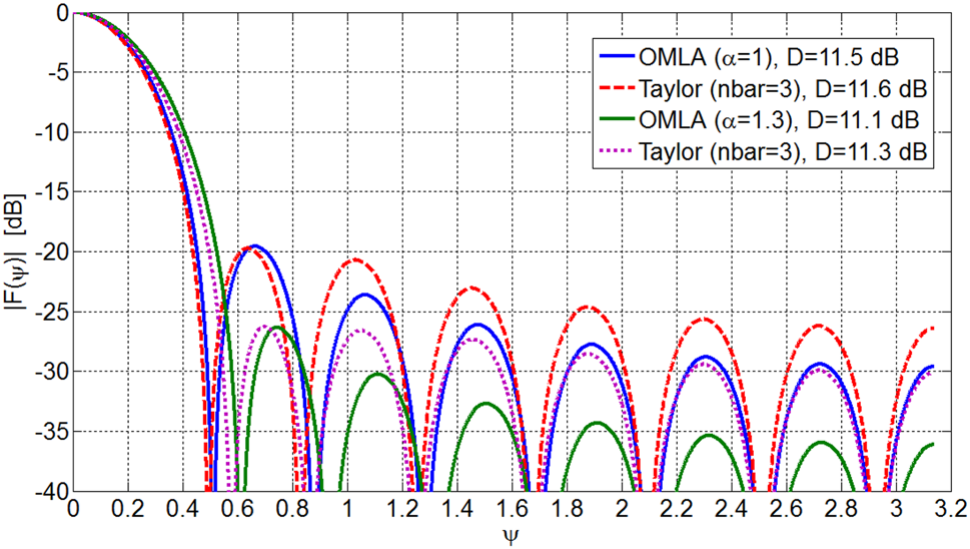
Array factors and directivity of OMLA and Taylor of the same SLL for *L* = 15 elements and *d*/*λ* = 0.5.

**Figure 13:**
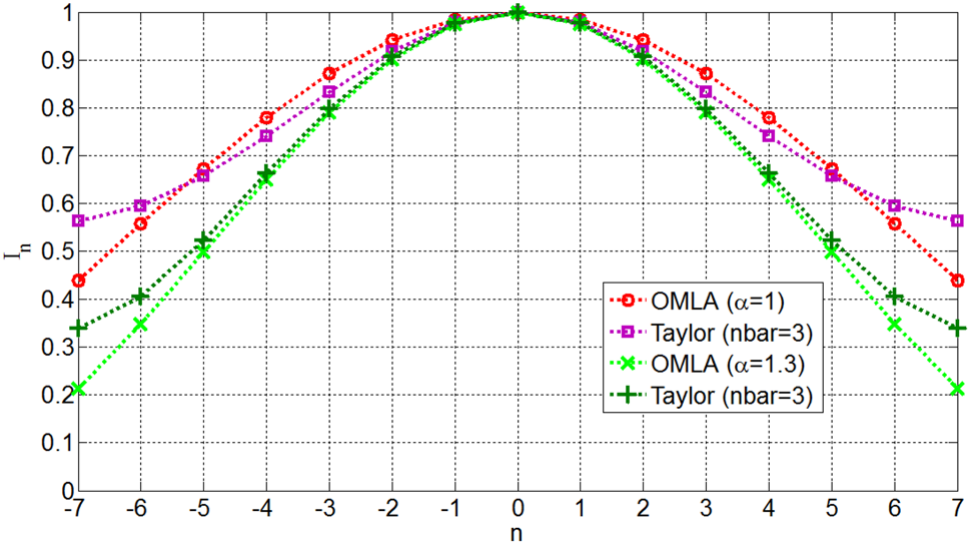
Excitation currents of elements for OMLA and Taylor patterns of *L* = 15 elements.

One can investigate that directivity of OMLA pattern is close to that of Taylor-nbar pattern^2^. Figure 12 compares these two patterns of the same SLL as well as directivity for *L* = 15 elements and *d*/*λ*  = 0.5. Also, Fig. 13 shows the excitation currents of the elements for these two types of patterns. It is seen that level of the second sidelobes onwards of OMLA pattern are less than those of Taylor pattern. Instead, the beamwidth of OMLA pattern is slightly greater than that Taylor one. So, the directivity of these two patterns are almost the same.

**Figure 14:**
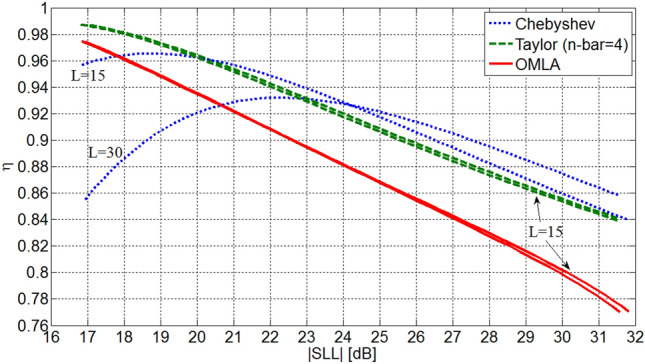
Taper efficiency of three types of patterns for *L* = 15 and 30.

The performance of OMLA can be compared with Chebyshev and Taylor patterns in terms of taper efficiency *η* = *D*/*D*_*u*_ where *D*_*u*_ is the directivity of uniform array. Figure 14 depicts taper efficiency of three patterns of *L* = 15 and 30 elements versus SLL for *d*/*λ*  = 0.5. The efficiency of OMLA pattern is slightly less than that of Taylor one but it is larger than the efficiency of Chebyshev pattern for |SLL| below a specified value.

The presented OMLA method has two important advantages over Taylor method. First, it gives us explicit relations for the excitation currents of elements by Eqs. (8) and (9). Second, the level of all sidelobes of OMLA pattern are equal or less than those of Taylor pattern for identical SLLs.

## Two dimensional Omla pattern

Usually, the pattern of planar arrays are equated as the multiplication of patterns of two linear arrays by supposing *I*_*mn*_ = *I*_*m*_*I*_*n*_^2^. The resultant two dimensional patterns would not have ring type sidelobes in *ψ*_*x*_-*ψ*_*y*_ plane. Here, we introduce a transformation to transform an arbitrary pattern of linear arrays, *F*(*ψ*), to the pattern of a planar array, *F*(*ψ*_*x*_,* ψ*_*y*_), having ring type sidelobes. The proposed transformation is as follows to apply the Eqs. (1) and (2).

14 $$ \psi = \sqrt {\psi_{x}^{2} + \psi_{y}^{2} } $$

where *ψ*_*x*_ and *ψ*_*y*_ are real variables defined as $\psi_{x} = 2\pi \frac{d}{\lambda }sin\theta \cos \varphi$ and $\psi_{y} = 2\pi \frac{d}{\lambda }sin\theta sin\varphi$. This transformation gives a more circular ring type pattern than the transformation presented in^16^.

The required excitation currents of planar arrays can be obtained from well-known methods such as two dimensional sampling method or Fourier's series method, like the following relation for odd by odd number of elements.

15 $$ I_{mn} = \frac{1}{{4\pi^{2} }}\int\limits_{ - \pi }^{\pi } {\int\limits_{ - \pi }^{\pi } {F(\psi_{x} ,\psi_{y} )\exp \left( { - j(m\psi_{x} ,n\psi_{y} )} \right)d\psi_{x} d\psi_{y} } } $$

**Figure 15:**
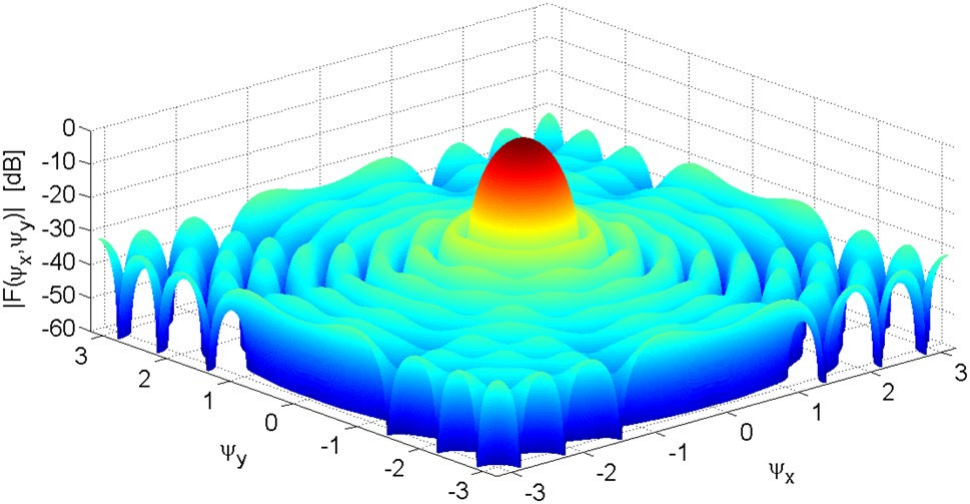
OMLA pattern of *α* = 1.3 for a planar array of 15 by 15 elements.

**Figure 16:**
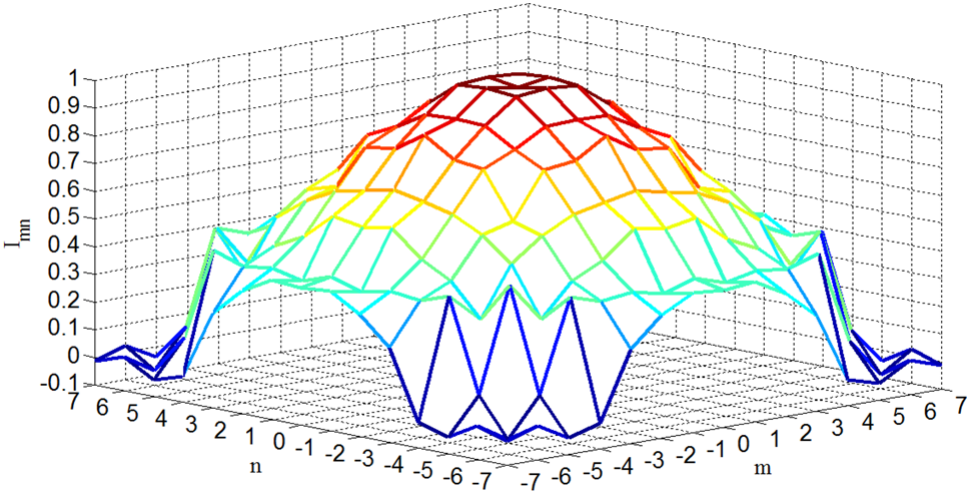
Excitation currents of elements for OMLA pattern of *α* = 1.3 for a planar array of 15 by 15 elements.

Figure 15 shows the OMLA pattern of *α* = 1.3 for a planar array of 15 by 15 elements. The ring type sidelobes are seen obviously. Also, Fig. 16 shows the excitation currents which have ring type symmetry and create an almost circular boundary planar array.

## Conclusion

An analytic method was proposed to design uniformly spaced arrays so that have as low as possible sidelobe level and having directivity as close as to that of uniformly excited arrays. The proposed method gives us explicit relations for the excitation currents. The synthesized array would have sidelobe levels which can be controlled by the expansion factor which is related to the beamwidth of the main lobe of the ideal desired array factor. It was seen that as the expansion factor increases the SLL decreases at the expense of some reduction in the directivity and some increase in the HPBW. The second sidelobes onwards of the synthesized pattern are less than those of Taylor pattern while the directivity of these two patterns are almost the same.
